# Supplementary material for: Streptococcus pneumoniae serotype 3 population structure in the era of conjugate vaccines, 2001–2018
Source: Microb Genom. 2024 Mar 18;10(3):001196. doi: 10.1099/mgen.0.001196 (PMC10963907; doi:10.1099/mgen.0.001196)

Supplemental Material

Streptococcus pneumoniae serotype 3 population structure in the era of conjugate vaccines, 2001-2018

Eleonora Cella[1], Catherine G Sutcliffe[2], Lindsay R Grant[2], Carol Tso[2], Robert C Weatherholtz[2], Shea Littlepage[2], Ladonna Becenti[2], Mohammad Jubair[1], Brenna C Simons[3], Marcella Harker-Jones[3], Raymond Reid[2], Del Yazzie[4], Mathuram Santosham[2], Katherine L O'Brien[2], Laura L Hammitt[2*], Taj Azarian[1*]

1 Burnett School of Biomedical Sciences, University of Central Florida, Orlando, Florida
2 Center for Indigenous Health, Johns Hopkins Bloomberg School of Public Health, Baltimore, Maryland
3 Arctic Investigations Program, Centers for Disease Control and Prevention, Anchorage, Alaska
4 Navajo Epidemiology Center, Window Rock, Arizona

Current affiliations: LRG – Pfizer; MJ- icddr,b; KLOB – World Health Organization
*Contributed equally

Supplemental Table 1. Characteristics of cases with serotype 3 invasive pneumococcal disease, Navajo Nation, 2001-2018

	**Total (n=131)**	**2001-2005 (n=21)**	**2006-2010 (n=29)**	**2011-2015 (n=37)**	**2016-2018 (n=44)**	**p-value**
Sex – female	55 (42.0%)	10 (47.6%)	12 (41.4%)	15 (40.5%)	18 (40.9%)	1
Age group (years)						0.008
<5	14 (10.7%)	5 (23.8%)	6 (20.7%)	1 (2.7%)	2 (4.5%)	
5-17	0 (0%)	0 (0%)	0 (0%)	0 (0%)	0 (0%)	
18-49	25 (19.1%)	3 (14.3%)	8 (27.6%)	7 (18.9%)	7 (15.9%)	
50-64	46 (35.1%)	4 (19.0%)	12 (41.4%)	12 (32.4%)	18 (40.9%)	
≥65	46 (35.1%)	9 (42.9%)	3 (10.3%)	17 (45.9%)	17 (38.6%)	
Source of isolate						1
Blood	126 (96.2%)	21 (100.0%)	28 (96.6%)	35 (94.6%)	42 (95.5%)	
Cerebrospinal fluid	2 (1.5%)	0 (0%)	0 (0%)	1 (2.7%)	1 (2.3%)	
Pleural fluid	3 (2.3%)	0 (0%)	1 (3.4%)	1 (2.7%)	1 (2.3%)	
Clinical syndrome[a]						
Pneumonia	112 (85.5%)	15 (71.4%)	25 (86.2%)	32 (86.5%)	40 (90.9%)	0.248
Meningitis[b]	5 (3.8%)	1 (4.8%)	0 (0%)	2 (5.4%)	2 (4.5%)	0.665
Non pneumonia/ meningitis	17 (13.0%)	5 (23.8%)	4 (13.8%)	5 (13.5%)	3 (6.8%)	0.281
PCV13 doses among children <5 years in 2011-2018 (n=3)						1
≥3	3 (100%)	n/a	n/a	1 (100.0%)	2 (100.0%)	
PPV23 and PCV13 doses among adults ≥50 years (n=92)						0.001
0	25 (27.2%)	4 (30.8%)	8 (53.3%)	6 (20.7%)	7 (20.0%)	
≥1 PPSV23 only	44 (27.8%)	8 (61.5%)	3 (20.0%)	20 (69.0%)	13 (37.1%)	
≥1 PCV13 only	1 (1.1%)	n/a	n/a	0 (0%)	1 (2.9%)	
≥1 PPSV23 and PCV13	11 (12.0%)	n/a	n/a	0 (0%)	11 (31.4%)	
Unknown	11 (12.0%)	1 (7.7%)	4 (26.7%)	3 (10.3%)	3 (8.6%)	
Any underlying medical conditions[c]	100 (76.3%)	12 (57.1%)	21 (72.4%)	26 (70.3%)	41 (93.2%)	0.003
Hospitalized[d]	125 (97.7%)	20 (100%)	26 (89.7%)	37 (100%)	42 (100%)	0.014
Outcome – died[e]	15 (12.5%)	5 (25.0%)	2 (7.4%)	2 (6.1%)	6 (14.3%)	0.190

n/a: not applicable; PCV: pneumococcal conjugate vaccine; PPSV: pneumococcal polysaccharide vaccine

[a] Individuals may have been diagnosed with more than one syndrome

[b] Three cases presenting with meningitis had blood but not cerebrospinal fluid collected for culture

[c] Underlying conditions assessed by medical chart review and restricted to those identified by the Advisory Committee on Immunization Practices (ACIP) as warranting administration of pneumococcal vaccines [64]

[d] Admission status missing for 1 case in 2001-2005

[e] Outcome missing for 11 cases (1 in 2001-2005; 2 in 2006-2010; 4 in 2011-2015; 4 in 2016-2018)

Supplemental Table 2. Characteristics of participants with serotype 3 carriage, Navajo Nation, 2006-2018

	**Total (n=61)**	**2006-2010 (n=34)**	**2011-2015 (n=14)**	**2016-2018 (n=13)**	**p-value**
Sex – female	28 (45.9%)	18 (52.9%)	5 (35.7%)	5 (38.5%)	0.510
Age group (years)					0.070
<5	43 (70.5%)	26 (76.5%)	6 (42.9%)	11 (84.6%)	
5-17	6 (9.8%)	4 (11.8%)	2 (14.3%)	0 (0%)	
18-49	9 (14.8%)	4 (11.8%)	4 (28.6%)	1 (7.7%)	
50-64	1 (1.6%)	0 (0%)	1 (7.1%)	0 (0%)	
≥65	2 (3.3%)	0 (0%)	1 (8.1%)	1 (7.7%)	
PCV13 doses among children <5 years (n=17)					0.052
0	2 (11.8%)	n/a	1 (16.7%)	1 (9.1%)	
1-2	5 (31.3%)	n/a	3 (50.0%)	2 (18.2%)	
≥3	10 (62.5%)	n/a	2 (33.3%)	8 (72.7%)	
PPV23 and PCV13 doses among adults ≥50 years (n=3)					1
0	2 (66.7%)	0 (0%)	1 (50.0%)	1 (100.0%)	
≥1 PPSV23 only	1 (33.3%)	0 (0%)	1 (50.0%)	0 (0%)	
≥1 PCV13 only	0 (0%)	n/a	0 (0%)	0 (0%)	
≥1 PPSV23 and PCV13	0 (0%)	n/a	0 (0%)	0 (0%)	

n/a: not applicable; PCV: pneumococcal conjugate vaccine; PPSV: pneumococcal polysaccharide vaccine

Supplemental Table 3. Vaccination history by clade and age among cases with serotype 3 invasive pneumococcal disease, 2001-2018

	Clade II	***Clade Iα***	**Non-CC180 clade**	**p-value**
**Children**				
**2011-2018**				
N	1	2	0	
Received any PCV13 doses, n (%)	1 (100%)	2 (100%)	n/a	---
**Adults**				
**2001-2010**				
N	7	13	12	
Received any PPSV23 doses, n (%)	2 (29%)	5 (38%)	4 (33%)	0.90
**2011-2018**				
N	48	11	8	
Received any PCV13/PPSV23 doses, n (%)	35 (73%)	7 (64%)	7 (87%)	0.51

n/a: not applicable; PCV: pneumococcal conjugate vaccine; PPSV: pneumococcal polysaccharide vaccine

Supplemental Figure 1. Incidence of serotype 3 invasive pneumococcal disease in adults (top) and children (bottom), Navajo Nation, 2000-2019

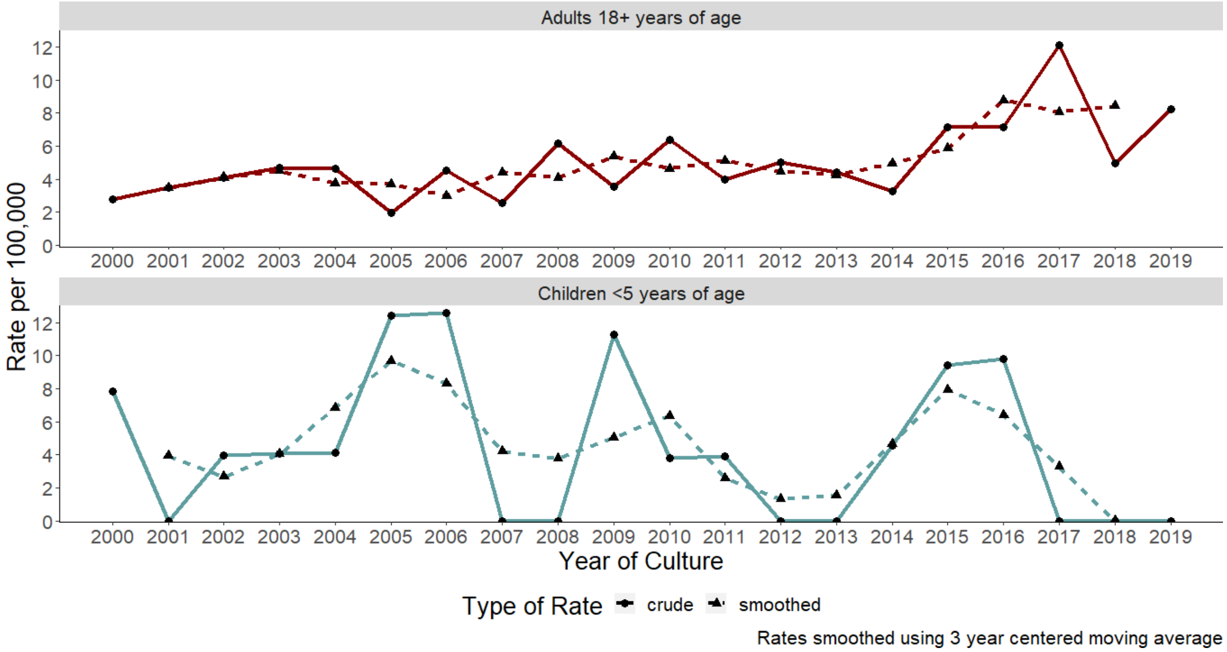

Supplement: Uncited Supplementary Material 1. [file mgen-10-01196-s001.pdf]
